# Disentangling stimulus and response compatibility as potential sources of backward crosstalk

**DOI:** 10.3758/s13414-020-02039-6

**Published:** 2020-06-11

**Authors:** Tobias Rieger, Jeff Miller

**Affiliations:** 1 Department of Psychology and Ergonomics, Chair of Work, Engineering, and Organizational Psychology, Technische Universität Berlin, Marchstraße 12, 10587 Berlin, Germany; 2 Department of Psychology, University of Otago, PO Box 56, Dunedin, 90654 New Zealand

**Keywords:** Backward crosstalk effect, Dual-tasking, Stimulus compatibility, Response compatibility, Flanker task

## Abstract

In two experiments (*N*= 60 each), we investigated the locus of backward crosstalk effects in dual tasking. Specifically, we embedded the typical flanker task within a dual-task paradigm by assigning stimulus-response (S-R) rules to the flankers. In Experiment 1, participants were instructed to first respond to the center letter and only respond to the flanker if the center was a no-go stimulus (i.e., prioritized processing paradigm). Mapping condition was varied between-subjects to be either matched (i.e., same S-R rule for flankers as for center letters), reversed (i.e., opposite S-R rule for flankers), or neutral (i.e., different letters for flankers with separate S-R rules). The results indicated that the backward crosstalk effect was mainly driven by a stimulus-based compatibility, as indicated by a significant S_2_−R_1_ compatibility effect in the matched and reversed conditions, with little change in this effect between the matched and reversed conditions. Experiment 2 replicated and extended these findings to a psychological refractory period paradigm. The present findings suggest that in the matched and reversed conditions, there was only one S-R rule active at a time.

A plethora of everyday life situations involves having more than one task at hand (i.e., multitasking; Koch, Poljac, Müller, & Kiesel, 2018). One special case of multitasking situations is having to work on two tasks simultaneously (i.e., dual tasking). In a laboratory setting, participants in dual-task situations are often required to respond to two tasks in rapid succession and the general finding is that performance for both tasks suffers compared to a single-task situation (Pashler, 1984; 1994; Tombu & Jolicoeur, 2004), even when only one overt response per trial is required and the other task requires no response (Miller & Durst, 2014; 2015). Another well-documented finding is that when the second task (T_2_) characteristics (e.g., stimuli or responses) are incompatible with the characteristics of the first task (T_1_), this typically leads to interference, and is often termed a backward crosstalk effect. In dual-tasking studies, it has often been shown that characteristics of T_2_ can influence first task performance (e.g., Caessens, Hommel, Reynvoet, & Vandergoten, 2004; Ellenbogen & Meiran, 2008; Hommel, 1998; Huestegge, Pieczykolan, & Janczyk, 2018; Janczyk, Renas, & Durst, 2018; Ko & Miller, 2014; Lien & Proctor, 2000; Lien, Ruthruff, Hsieh, & Yu, 2007; Logan & Schulkind, 2000; Miller, 2006; Miller & Durst, 2015; Navon & Miller, 1987).

## Backward crosstalk effects

Navon and Miller (1987) found that processing of two tasks rarely occurs independently, and this potentially allows for interference from one task to the other. Most interestingly, they found that characteristics of a task performed later could influence performance of a task performed earlier, which might be termed a “backward crosstalk effect” (BCE). In a seminal study of BCEs, Hommel (1998) used stimuli with two dimensions (e.g., a colored letter), and the two tasks required sequentially responding to both dimensions. One central finding of this study was that when the response to the second task (R_2_) was compatible with the response to the first task (R_1_) (e.g., first having to respond with the left hand to the stimulus’ color, R_1_, and then responding with a vocal “left” to the stimulus’ identity, R_2_), responses to the first task were faster than when R_2_ was incompatible with R_1_ (e.g., first having to respond with the left hand as R_1_ and the verbally responding “right” as R_2_). The general finding of this and other types of backward crosstalk has been replicated and extended in various studies (e.g., Hommel and Eglau, 2002; Janczyk, 2016; Logan & Schulkind, 2000).

One reason why the existence of BCEs in dual-task situations has puzzled researchers is that it challenges the assumption that only one stimulus-response (S-R) rule can be active at a time during the response selection stage of processing, which is a key assumption of response selection bottleneck (RSB) models (e.g., Pashler, 1994). Hommel (1998) argued that S-R translation can occur automatically and is a distinct stage from final response selection—and therefore also allows for parallel activation of a given response by more than one S-R rule at a time. A different theoretical account integrates BCEs into resource sharing models, such as the EPIC model by (Meyer and Kieras, 1997a; 1997b). Specifically, the EPIC model (along with other resource sharing models) has no limitation in how many S-R rules can be active at a time—even for response selection. Thus, the main difference in accounting for BCEs in RSB models and resource sharing models is that in the former, responses are automatically activated upon stimulus presentation as a process which is distinct from response selection and in the latter, activation of incompatible responses can occur during the response selection stage.

Besides interference effects of task-relevant characteristics (e.g., interfering activation of different response sets of the second task), it has also been shown that task-irrelevant stimulus and response features can produce crosstalk. For instance, Miller and Alderton (2006) found that instructed response force in the second task affected the response force to a first task, without any instructions to modulate response force to T_1_. Moreover, Ruiz Fernández and Ulrich (2010) found that T_2_ movement distance to execute the second response influenced the RTs to the first task, even though T_1_ just required a key press—not a ballistic movement.

## The present experiments

The present experiments aimed at disentangling the separate contributions of two different sources of compatibility to the BCEs observed in paradigms with common response sets for T_1_ and T_2_. Our general approach followed a logic similar to that of Janczyk, Pfister, Hommel, and Kunde (2014). In their experiments, they aimed at disentangling response compatibility effects from action effect compatibility effects. To this end, they used a dual-task paradigm where each task was mapped to one hand. Specifically, participants were always asked to first respond to T_1_ and subsequently to T_2_, resembling a psychological refractory period (PRP) paradigm, but without varying the stimulus onset asynchrony (SOA) between S_1_ and S_2_. Importantly, in two conditions, the response to T_2_ could result in either compatible or incompatible action effects (i.e., a light appearing or a virtual lever moving toward either the same or opposite direction as the required response side). Their main finding was that the effect of R_2_−R_1_ compatibility (e.g., left-left responses as compatible) was significantly decreased—and even descriptively reversed (i.e., negative R_2_−R_1_ compatibility effect)—if the associated action effects were incompatible. Thus, they interpreted their findings as indicating that action effect compatibility plays a considerable role in the emergence of BCEs.

The present study aims at an analogous investigation of possibly separate contributions of stimulus and response compatibility to the BCE. Two major—not entirely mutually exclusive—possible accounts of BCEs are: (a) stimulus-based compatibility, i.e., application of the T_1_ rule to S_2_ creates interference if the stimulus of T_2_ is associated with an incompatible response of T_1_ (S_2_−R_1_ compatibility), and (b) response-based compatibility, i.e., the response to T_2_ is activated in time and influences T_1_ processing (R_2_−R_1_ compatibility). Crucially, the S_2_−R_1_ compatibility account of BCEs is in principle compatible with bottleneck models, as only one response is being selected at a time, even though multiple stimuli are driving that response—with only one S-R rule being active at that time. Moreover, S_2_−R_1_ compatibility could in principle also just be a case of a single response being driven by more than one source of information, as is typically observed in the flanker task (B. A. Eriksen & Eriksen 1974) and in experiments investigating coactivation (e.g., Miller, 1982). On the other hand, the R_2_−R_1_ compatibility account, which is presumably mediated by motor activation (e.g., Ko & Miller, 2014; Lien et al., 2007; Miller, 2017), is incompatible with pure RSB models of dual-task processing, since multiple S-R rules are being used to activate responses simultaneously.

To discriminate between these accounts, we embedded the flanker task (Eriksen & Eriksen, 1974) within a dual-task paradigm by assigning an S-R rule not just for the center letter, but also for the flankers. In the classic flanker task, participants are presented a string of letters (e.g., KKSKK) and are instructed to only respond to the identity of the center letter, ignoring the (irrelevant) identity of the flanker letters. In this task, the flanker letters usually produce compatibility effects. Specifically, when the flanker letters are assigned to a different response than the center letter, responses are slower than when the flanker letters are assigned to the same response as the center letter (e.g., B. A. Eriksen & Eriksen, 1974; C. W. Eriksen & Schultz, 1979; Miller, 1991).

In our present experiments, we used the general flanker set-up to investigate the source of dual-task BCEs. To separate S_2_−R_1_ compatibility from R_2_−R_1_ compatibility as potential sources of the BCE, it seems necessary to have comparable stimuli and responses for the two tasks, and using letter tasks as T_1_ and T_2_ in a flanker dual-task setup seems ideal for this purpose. In Experiment 1, we used a prioritized processing (PP) paradigm (Miller & Durst, 2014), in which participants are asked to first respond to the center letter as quickly and accurately as possible and to respond to the flanker letters only if the center letter requires a no-go response. In Experiment 2, we embedded the flanker task in a PRP paradigm (Telford, 1931; Welford, 1952) where participants were always asked to first respond to the center letters and then respond to the flanker letters (somewhat similar to the paradigm used by Hübner & Lehle, 2007, and Lehle & Hübner, 2009), potentially leading to stronger compatibility effects than in the PP paradigm (Miller & Durst, 2015; Mittelstädt & Miller, 2017).

The key manipulation in both experiments was the S-R rule assigned to the flanker letters (i.e., Task 2), as is illustrated in Table 1. Specifically, we used three different flanker S-R rules and varied the rules between-subjects in order to avoid any possible confusions with having to change the S-R rule throughout the experiment. In the *matched* mapping condition, the S-R rule used for the flankers was identical to the S-R rule used for the center letter. For instance, if the letter “B” required a left key press when it appeared in the center, it also required a left key press when it appeared as a flanker. Consequently, in the matched condition, trials that were R_2_−R_1_ compatible were also S_2_−R_1_ compatible. For the example in Table 1, responses to the center target B in the stimulus CCBCC should be quite fast in the matched condition, because the flanker C is assigned to the same response as the target B within both S-R rules. Thus, the response would be facilitated both by S_2_−R_1_ activation and by R_2_−R_1_ activation. In the *reversed* mapping condition, the S-R rule used for the flankers was reversed relative to the S-R rule for the center letter. For example, if the letter “B” required a left key press in the center location, it would require a right key press appearing as a flanker. Thus, in the reversed condition, trials which are S_2_−R_1_ compatible are automatically R_2_−R_1_ incompatible. For the example in Table 1, responses to the center target B in the stimulus CCBCC should not be particularly fast in the reversed condition, because the flanker C is assigned to the opposite response within the T_2_ S-R rule. Thus, the response would be facilitated by S_2_−R_1_ activation but inhibited by any R_2_−R_1_ activation that is present. The third condition was the *neutral* mapping condition. Here, two distinct letter sets were used for the flankers and the center letters, and the two sets thus had separate S-R rules. Consequently, there were no cases of S_2_−R_1_ compatible trials in the neutral condition, and R_2_−R_1_ compatibility was the only possible source of compatibility effects. The neutral condition mainly served as a control condition with only R_2_−R_1_ compatibility, without S_2_−R_1_ compatibility.

**Table 1:** Exemplary stimulus-response rules and associated compatibility types for the flanker dual task

Task 1 S-R Rule		Task 2 S-R Assignment Group & S-R Rule					
		Matched		Reversed		Neutral	
Stimuli	Response	Stimuli	Response	Stimuli	Response	Stimuli	Response
B,C	Left	B,C	Left	B,C	Right	K,L	Left
X,Y	Right	X,Y	Right	X,Y	Left	M,N	Right
Sample stimulus		Types of compatibility in each condition					
CCBCC		S_2_ compatible with R_1_		S_2_ compatible with R_1_			
		R_2_ compatible with R_1_		R_2_ incompatible with R_1_			
XXBXX		S_2_ incompatible with R_1_		S_2_ incompatible with R_1_			
		R_2_ incompatible with R_1_		R_2_ compatible with R_1_			
LLBLL						S_2_ neutral with R_1_	
						R_2_ compatible with R_1_	
NNBNN						S_2_ neutral with R_1_	
						R_2_ incompatible with R_1_	

Note that in the actual experiment, letters were randomly assigned to the responses and not grouped in adjacent letters as is the case in this example. S: stimulus, R: response

**Fig. 1:**
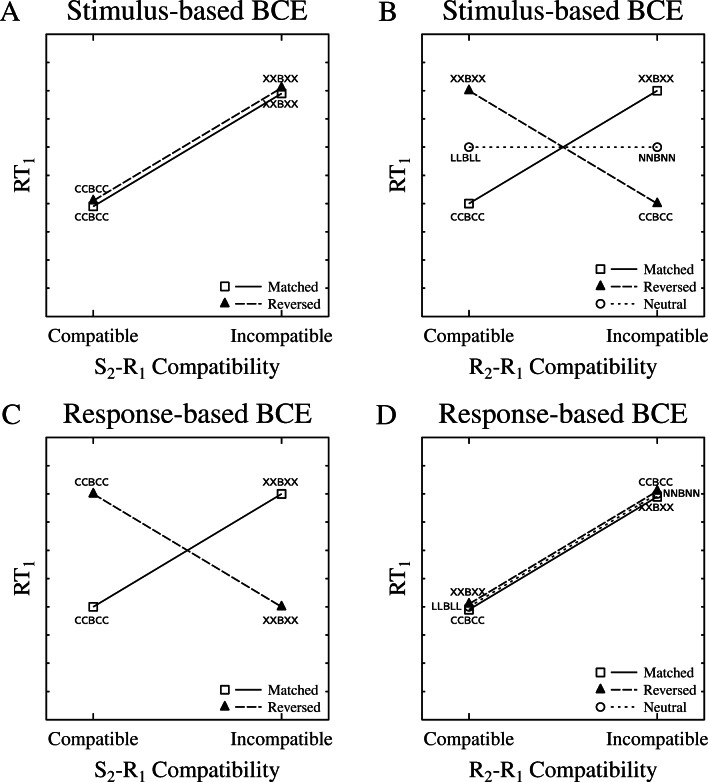
Theoretical predictions of two models regarding the source of the BCE. The predictions of both models are displayed twice in the present figure, once as a function of stimulus compatibility (S_2_−R_1_; A and C), and once as a function of response compatibility (R_2_−R_1_; B and D). A and B: predictions if the BCE is purely stimulus-based. C and D: predictions if the BCE is purely response-based. See main text for explanation of the predictions. Because there was no S_2_−R_1_ compatibility in the neutral condition (see Table 1), this condition is not included in panels A and C. RT: reaction time, S: stimulus, R: response, BCE: backward crosstalk effect

Figure 1 visualizes predictions for this experiment for the extreme cases in which BCEs are driven exclusively by S_2_−R_1_ compatibility (A and B) or exclusively by R_2_−R_1_ compatibility (C and D). Panels A and C plot the possible results in terms of S_2_−R_1_ compatibility; thus, in the example of Table 1, CCBCC would be classified as S_2_−R_1_ compatible and XXBXX would be classified as S_2_−R_1_ incompatible for both the matched and the reversed conditions. This is because—with compatibility defined in terms of the T_1_ S-R rule—the flankers C and X are associated with the matching and mismatching R_1_’s, respectively. Thus, if BCEs are driven exclusively by S_2_−R_1_ compatibility (A), responses should be faster in the S_2_−R_1_ compatible condition than in the S_2_−R_1_ incompatible condition, and this compatibility effect should be the same for both the matched and the reversed conditions (A). On the other hand, as is shown in Panel C, the pattern should look quite different in the reversed condition if BCEs are driven exclusively by R_2_−R_1_ compatibility. With this type of compatibility and the reversed T_2_ mapping condition, the response to the B in XXBXX would be relatively fast because the T_2_ S-R rule associates X with the same response that is required in T_1_ for B. Furthermore, the response to the B in CCBCC would be relatively slow because the T_2_ S-R rule associates C with the opposite response. Thus, if the BCE is driven by R_2_−R_1_ compatibility, the S_2_−R_1_ compatibility effect should invert in the reversed condition (C). Panels B and D re-plot the same results with trials classified in terms of R_2_−R_1_ compatibility, which changes the compatible/incompatible classification of trials in the reversed condition. Thus, in the example of Table 1, XXBXX would be classified as R_2_−R_1_ compatible because X is associated with the correct R_1_ within the T_2_ S-R rule. Similarly, CCBCC would now be classified as incompatible because C is associated with the incorrect R_1_ within the T_2_ S-R rule. With compatibility classified in this way, there should be a positive compatibility effect if the BCE is driven by R_2_−R_1_ compatibility (D), but a negative compatibility effect if the BCE is driven by S_2_−R_1_ compatibility (B). Note that there is no such thing as S_2_−R_1_ compatibility in the neutral condition, because the flanker letters in this condition are not associated with responses within the T_1_ S-R rule, so the neutral condition cannot be plotted in (A) and (C). There is still R_2_−R_1_ compatibility in the neutral condition, however, since the flankers are associated with responses in the T_2_ S-R rule. There would be no R_2_−R_1_ compatibility effect in this condition if the BCE is entirely stimulus-driven (B). If the BCE is entirely driven by R_2_−R_1_ compatibility, however, there would be the same positive BCE in the neutral condition as in the other two conditions (D).

In summary, the present experiments therefore allow us to disentangle stimulus (S_2_−R_1_) and response (R_2_−R_1_) compatibility, with S_2_−R_1_ compatibility referring to the idea that applying the T_1_ rule to S_2_ tends to activate R_1_ according to that rule. If the flankers directly activate R_2_ in time, the S_2_−R_1_ compatibility effect should be smaller in the reversed condition than in the matched condition—or possibly even reversed (i.e., a negative S_2_−R_1_ compatibility effect and a positive R_2_−R_1_ compatibility effect in the reversed condition). Conversely, if the BCE is mainly stimulus driven (i.e., only one S-R rule is active at a time), one should find no differences in the size of the S_2_−R_1_ compatibility effects between the matched and reversed conditions—and, consequently, a negative R_2_−R_1_ compatibility effect in the reversed condition. The neutral condition mainly served as a control condition with only R_2_−R_1_ compatibility, without S_2_−R_1_ compatibility.

## Experiment 1

As mentioned above, in Experiment 1, the flanker task was embedded in a PP paradigm (Miller & Durst, 2014). In the present adaptation of the PP paradigm, participants are asked to first respond to the center letter, and only respond to the flanker letter if the center letter requires a no-go response. Consequently, any trial that requires a response to the center letter, ends after that response is executed, resembling the classic flanker task with just one overt response per trial. The PP paradigm shares many observable commonalities with the PRP paradigm (Miller & Durst, 2015), and also BCEs have been found in the PP paradigm (e.g., Miller & Durst, 2015; Mittelstädt & Miller, 2017). This therefore allows us to use this paradigm to investigate the BCEs that are typically present in dual-task situations, and try to further locate the sources of this BCE.

One previous study which investigated the BCE in the PP paradigm in more detail was Miller (2017). In this study, lateralized readiness potentials were used to determine the time at which BCEs arise during processing. The results suggested that BCEs are present because T_2_ stimuli influence T_1_ response selection (i.e., S_2_−R_1_ compatibility effect)—and not because T_2_ stimuli activate their corresponding R_2_ (i.e., no evidence for R_2_−R_1_ compatibility). The present experiment, then, aims at further shedding light on the nature of BCEs using a dual-task flanker paradigm, and at separating these two kinds of compatibility effects. Specifically, as mentioned above, we used three mapping conditions (i.e., matched: S_2_−R_1_ compatible trials were also R_2_−R_1_ compatible; reversed: S_2_−R_1_ compatible trials were R_2_−R_1_ incompatible; and neutral: S_2_ neutral to R_1_, and separate R_2_−R_1_ compatibility) to separate the contributions S_2_−R_1_ and R_2_−R_1_ compatibility.

### Method

#### Participants

Participants were 60 University of Otago psychology students (46 women) who took part in the experiment in exchange for course credit. They ranged in age from 17 to 26 (*M*= 19.8) and they were predominantly right-handed (*M* = 59.6) as indexed by the Edinburgh Handedness Inventory (Oldfield, 1971). We planned on sample sizes of 20 participants with accuracy above 80% per S-R mapping condition but actually tested one extra participant in the matched and neutral conditions due to the unpredictabilities associated with experimental participation. To obtain equal sample sizes in each condition, we dropped the participant with the lowest accuracy from the matched and neutral groups. We further excluded six additional participants due to low accuracy (i.e., below 80%).

#### Apparatus and stimuli

The experiment took place in individual test rooms. Stimulus presentation and recording of responses were controlled by an IBM-PC compatible computer using MATLAB with the Psychophysics Toolbox extension (Brainard, 1997; Pelli, 1997). Viewing distance was approximately 60 cm but not restrained. Stimuli were presented vertically and horizontally centered on a 17” screen in a white 35 point font—that is, a center letter was displayed flanked by two outside letters on each side. The letter stimuli were presented in white 35pt font. A centered, white plus sign (+) served as fixation point. Responses were key presses with the left and right index fingers on the “Z” and “?/” keys of a standard computer keyboard.

For each participant the letter stimuli were randomly selected from all consonants, excluding the letters L, R, and Z in order to avoid associations with response side or key. More specifically, two letters each were assigned for any of the three T_1_ response possibilities (i.e., left/right keypress, no-go). In the matched and reversed conditions, the same letters were used for T_2_ as for the go-stimuli for T_1_, with the same or reversed S-R rule, respectively. The T_1_ no-go letters never appeared as flankers in any condition. In the neutral condition, two additional stimuli were assigned to each possible response (i.e., left/right keypress) for T_2_, leading to a total of ten different letters used in this condition, and six different letters in the matched and reversed conditions. We only tested trials with different letters for the center and flanker stimuli, at least one of which was assigned to a go response, thus leaving 20 different trial types (we omitted one response-compatible stimulus combination in the neutral condition in order to obtain the same number of trial types in this condition).

#### Procedure

The single experimental session lasted approximately 45 min. Each subject was tested in one of the three conditions (i.e., matched, reversed, or neutral). The experiment consisted of two practice blocks and eight experimental blocks. The two practice blocks served as single-task training for T_1_ and T_2_, respectively, displaying the typical trial sequences but instructing participants to pay attention to only one of the two presented stimuli. In the experimental blocks, subjects were instructed to treat the center letter as the high priority task, and the flanker letters as the low priority task. That is, subjects were instructed to first respond to T_1_ with left/right index finger presses, respectively, and to only respond to T_2_ if S_1_ was the no-go stimulus. In the matched condition, the S-R rule was the same for T_2_ as for T_1_. In the reversed condition, the S-R rule for T_2_ was reversed to the rule for T_1_—that is, the stimuli which required a left response for T_1_ required a right response for T_2_ and vice versa. In the neutral condition, four additional letters served as the stimuli for T_2_ with two letters each corresponding to left/right responses, respectively. The mapping is illustrated in Table 1.

Each trial type was presented twice in the practice blocks (40 trials). The experimental blocks included 72 trials. In two-thirds of these trials, T_1_ required a response, and in one-third, T_2_ required a response. Of the T_1_ trials, half of the trials were R_2_−R_1_ compatible, and the other half were R_2_−R_1_ incompatible. In the trials where T_1_ required no response, half of the responses to T_2_ required a left-hand response and half required a right-hand response.

The trial sequence started with a fixation cross for 500 ms. The center letter and the flankers were displayed with an SOA of 100 ms. Stimuli remained on screen for a maximum of 2 s or until a response was given. Erroneous responses resulted in an error feedback screen display of 3 seconds. After responses faster than 200 ms or slower than 2 s, subjects were informed that they had responded too fast or too slow, respectively, for 3 s. If subjects made more than three consecutive errors, they were shown the instructions again as a reminder. Trials were followed by an intertrial interval of 2 s.

### Results

Practice blocks and the first experimental block were excluded from any further analysis as training. Erroneous trials were removed from the RT analyses (8.7%). One and 34 trials were removed from the analyses as RT outliers based on lower and upper RT cutoffs of 200 ms and 2 s, respectively. The analyses comparing the matched and reversed conditions focus on stimulus compatibility. In the neutral mapping condition, there is no S_2_−R_1_ compatibility as the flanker letters are always drawn from a different letter set than the center letters—thus, for this condition, we refer to the R_2_−R_1_ compatibility.

#### Primary task: RT_1_ and PE_1_

##### Matched vs. reversed conditions

**Fig. 2:**
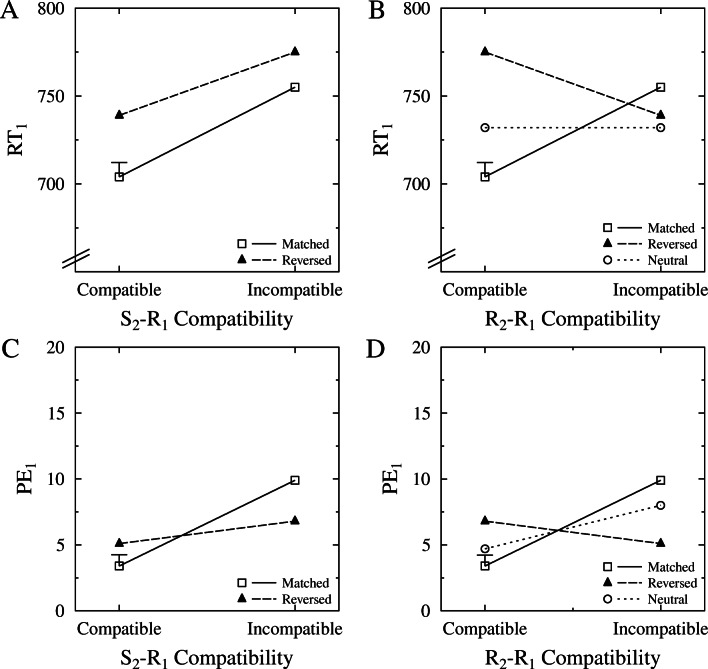
Experiment 1 results for the primary task as a function of both stimulus (i.e., S_2_−R_1_) compatibility (A, C, for RTs and percent errors) and response (i.e., R_2_−R_1_) compatibility (B, D, for RTs and percent errors), separately for each mapping condition. Note that as in the neutral condition, there was no S_2_−R_1_ compatibility and this condition is thus only present in the response compatibility plots. Please note that the figures on the right (B,D) replot the data from the figures on the left (A,C) referring to a different type of compatibility. Error bar represents the pooled standard error. RT: reaction time, PE: percent errors, S: stimulus, R: response

Figure 2A shows the means of RT_1_ for the matched and reversed mapping conditions as a function of S_2_−R_1_ compatibility. We ran an ANOVA with the between-subject factor condition (i.e., matched, reversed) and the within-subject factor of stimulus (i.e., flanker) compatibility for RT_1_. This ANOVA yielded a significant main effect of stimulus compatibility, *F*(1,38) = 51.119, *p* < .001, $\eta ^{2}_{p}=0.574$, with faster responses in stimulus compatible (721 ms) than in stimulus incompatible (765 ms) trials. Interestingly, no other effect was significant (*p* s >.241), indicating no differences in compatibility effects between the matched and reversed conditions.

We ran parallel analyses on the percentage of erroneous responses for the primary task (PE_1_). Figure 2B visualizes the results of this ANOVA. This ANOVA revealed a significant main effect of stimulus compatibility, *F*(1,38) = 45.690, *p* < .001, $\eta ^{2}_{p}=0.546$, with fewer erroneous responses in the stimulus compatible (4.3%) than in the stimulus incompatible (8.3%) trials. Interestingly, the condition x stimulus compatibility interaction was significant, $F(1,38) = 15.972, p < .001, \eta ^{2}_{p}=0.296$, indicating a larger stimulus compatibility effect in the matched (6.5%) than in the reversed (1.7%) condition, with the latter effect still significant, $F(1,19) = 7.175, p=.015, \eta ^{2}_{p}=0.274$. The main effect of condition was not significant, *p* =.519.

However, the interpretation of the PE data is not as straightforward in the PP paradigm, because R_1_ and R_2_ were both performed with the same response keys. It is therefore not possible to distinguish which of the two tasks the participant actually aimed to respond to, and there are most likely some trials where a participant incorrectly responded to T_2_ where s/he should have responded to T_1_. In the matched condition, the responses in these trials were correct in the S_2_−R_1_ compatible trials and incorrect in the reversed condition. Thus, these “wrong task” trials increase the S_2_−R_1_ BCE on PE in the matched condition. In the reversed condition, however, the opposite happens, and the “wrong task” trials decrease the compatibility effect. It might therefore be possible that this task confusion effect underlies the significant interaction reported above.

**Fig. 3:**
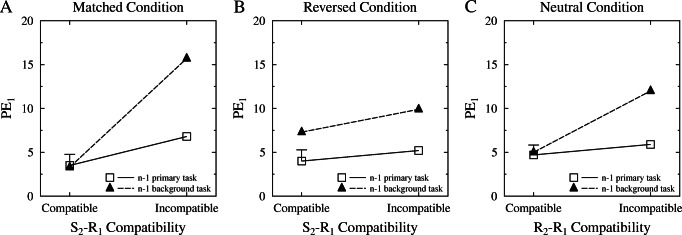
Percent errors (PE) as a function of S_2_−R_1_ compatibility (A and B for the matched and reversed conditions, respectively) and as a function of R_2_−R_1_ compatibility (C for the neutral condition), separately for trials following a trial where the previous (n-1) trial required a background task response or a primary task response. Error bar represents the pooled standard error. S: stimulus, R: response

We checked whether the task (i.e., T_1_ vs. T_2_) responded to on trial n-1 played a role here, because it is reasonable to assume that this task confusion happens more often following a background task response. We thus added the task on trial n-1 as an additional factor in the ANOVA reported above. Figure 3 visualizes the separate ANOVAs for each mapping condition when including the n-1 task factor. This ANOVA produced a significant three-way interaction of condition, stimulus compatibility, and n-1 task, $F(1,38) = 14.096, p=.001, \eta ^{2}_{p}=0.271$. We followed this up by separately running ANOVAs for each mapping condition. In the matched condition, there was a significant interaction between n-1 task and stimulus compatibility, $F(1,19) = 39.463, p < .001, \eta ^{2}_{p}=0.675$. In the reversed condition, this interaction was not significant, $F(1,19) = 0.955, p=.341, \eta ^{2}_{p}=0.048$. As can be seen in Fig. 3A and B, it seems as if the larger S_2_−R_1_ compatibility effect just stems from the trials following a background task response. Thus, it seems plausible that this task confusion was responsible for the difference in the sizes of the S_2_−R_1_ compatibility effects between the matched and reversed conditions.

##### Neutral condition

Figure 2C shows the means of RT_1_ for all groups (i.e., matched, reversed, neutral) as a function of R_2_−R_1_ compatibility, thus also showing the means of the neutral condition. In the neutral condition, we did not observe any R_2_−R_1_ compatibility effect, $F(1,19) < 0.01, p=.939, \eta ^{2}_{p}=0$, with no difference between R_2_−R_1_ compatible (732 ms) and incompatible (732 ms) trials. Separate ANOVAs with each pair of conditions showed that this null effect was significantly different from the compatibility effects obtained in both the matched condition, $F(1,38) = 20.275, p < .001, \eta ^{2}_{p}=0.348$, and the reversed condition, $F(1,38) = 10.486, p=.002, \eta ^{2}_{p}=0.216$.

We again ran parallel analyses on PE_1_, and Fig. 2D shows the means of PE_1_ for all three conditions as a function of R_2_−R_1_ compatibility. In the ANOVA of the neutral condition, responses in R_2_−R_1_ compatible trials were significantly less erroneous (4.7%) than in R_2_−R_1_ incompatible trials (8.0%), *F*(1,19) = 18.875, *p* < .001, ${\eta }^{2}_{p}=0.498$. Again, in the ANOVA comparing the neutral and matched conditions, there was a significant interaction of R_2_−R_1_ compatibility and condition (i.e., neutral vs. matched), *F*(1,38) = 6.315, *p* = .016, $\eta ^{2}_{p}=0.142$, indicating a smaller compatibility effect in the neutral (3.3%) than in the matched (6.5%) condition. In the ANOVA comparing the neutral and reversed conditions, the interaction of compatibility and mapping condition was not significant, *F*(1,38) = 2.705, *p* = .108, $\eta ^{2}_{p}=0.066$.

As was mentioned above, it is not possible to clearly distinguish which task a participant intended to respond to in the PP paradigm—possibly allowing task confusion effects in the error data to produce effects which look like compatibility effects. We therefore ran a parallel analysis including the n-1 task (i.e., T_1_ vs. T_2_) as an additional factor to the ANOVA reported above. The corresponding means of this ANOVA are displayed in Fig. 3C. There was a significant interaction of n-1 task and R_2_−R_1_ compatibility in the neutral condition, *F*(1,19) = 19.369, *p* < .001, $\eta ^{2}_{p}=0.505$, indicating that the R_2_−R_1_ compatibility effect for PE reported above is mainly due to trials following a background task response. Because there was no R_2_−R_1_ compatibility effect in the RT data, we believe that this task confusion effect is the most likely explanation for the R_2_−R_1_ compatibility effect in the PE data.

#### Background task: RT_2_ and PE_2_

An ANOVA with the between-subject factor mapping condition was run for RT_2_. We found a significant main effect of condition, *F*(2,57) = 16.422, *p* < .001, $\eta ^{2}_{p}=0.366$, with RT_2_ being fastest in the matched condition (828 ms), followed by the neutral condition (956 ms), and the slowest T_2_ responses in the reversed condition (1087 ms). Post hoc pairwise comparisons revealed significant differences between all three conditions (*p* s <.014). Note that it is not possible to examine effects on R_2_ of the match between R_1_ and R_2_ (i.e., forward compatibility) in the PP paradigm, because any trial with an R_2_ in the PP paradigm necessarily followed a no-go S_1_. Such effects will be examined in Experiment 2 using the PRP paradigm, however.

A parallel analysis was carried out for the percentage of erroneous responses for the background task (PE_2_). For PE_2_, a similar result pattern emerged. That is, the ANOVA revealed a significant main effect of condition, *F*(2,57) = 8.214, *p* = .001, $\eta ^{2}_{p}=0.224$. Post-hoc pairwise comparisons revealed a significant effect between the matched condition (9.9%) and the reversed condition (18.1%), *p* < .001, and between the neutral (12.6%) and the reversed condition, *p* = .015. The comparison between the matched and the neutral condition was not significant, *p* = .151.

### Discussion

The main findings of Experiment 1 can be summarized as follows. In both the matched and the reversed conditions, we found a significant S_2_−R_1_ compatibility effect, with little change in this effect across the two mapping conditions, replicating the general existence of BCEs in the PP paradigm (e.g., Miller, 2017; Miller and Durst, 2015; Mittelstädt & Miller, 2017). In the neutral condition—which featured only R_2_−R_1_ compatibility—we found no compatibility effect.^1^ Even though there was some evidence for an R_2_−R_1_ compatibility effect in the error data, we believe that this is mostly due to task confusion effects. This argument seems particularly plausible when considering that these R_2_−R_1_ compatibility effects stem only from trials that followed trials requiring a background task response—and it seems logical that this kind of task confusion (i.e., responding to T_2_ instead of T_1_) happens more often after responding to the background task.

The finding of a stimulus-based BCE on RT aligns well with previous findings in the PP paradigm. That is, it seems that the BCE is mainly based on S_2_−R_1_ compatibility, and not R_2_−R_1_ compatibility, aligning well with the findings of Miller (2017). S_2_−R_1_ compatibility seems to be the main source of the BCE in Experiment 1, as (a) the S_2_−R_1_ compatibility effect was also observed in the reversed condition, where S_2_−R_1_ compatible trials were R_2_−R_1_ incompatible, (b) this S_2_−R_1_ compatibility effect was not significantly decreased in the reversed compared to the matched condition, and (c) we did not observe any compatibility effect in the neutral condition, which only featured R_2_−R_1_ compatibility, but not S_2_−R_1_ compatibility. Moreover, there was also a main effect of mapping condition on RT_2_. Obviously, one would expect this effect because of mapping complications in the reversed and neutral conditions compared to the matched condition—however, our main concerns were regarding the BCEs in the different conditions.

However, as the PP paradigm only requires one overt response per trial, it is not clear whether that might have played a role in finding no evidence for an R_2_−R_1_ compatibility effect (or at least a decreased S_2_−R_1_ compatibility effect) in the reversed condition. Moreover, we also did not observe any R_2_−R_1_ compatibility effect in the neutral condition (which featured only R_2_−R_1_ but not S_2_−R_1_ compatibility), and this could also possibly have been due to the nature of the PP paradigm, where only one overt response per trial is required, with strong prioritization of T_1_. Therefore, Experiment 2 used a PRP paradigm—where on every trial two overt responses are necessary.

## Experiment 2

Previous studies comparing the PRP and the PP paradigms have found larger BCEs in the former paradigm (Miller and Durst, 2015; Mittelstädt & Miller, 2017). This finding makes sense because in the PP paradigm, participants usually prioritize the first task more strongly than in the PRP paradigm, possibly leading to less interference by T_2_ characteristics. Moreover, Rieger, Mittelstädt, Dignath, and Kiesel (2019) suggested that in the PRP paradigm, motor coordination possibly decreases flexibility in T_2_ processing compared to the PP paradigm, thus potentially also allowing for more direct R_2_−R_1_ interference. As previous studies using both the PP and the PRP paradigm all used unrelated tasks (i.e., letter and color classification tasks), they did not distinguish between different types of compatibility (i.e., S_2_−R_1_ and R_2_−R_1_), as we are aiming to do in the present experiments. In Experiment 2, we thus embedded the flanker task in a PRP paradigm. Participants were asked to always first respond as fast and as accurately as possible to the center letter and subsequently to respond as fast and as accurately to the flankers. Experiment 2 thus largely mirrored Experiment 1—with the main difference of a higher priority of T_2_ in the PRP paradigm than in the PP paradigm.

BCEs in the PRP paradigm have been extensively researched (for a review, see for example, Lien & Proctor, 2002). However, most studies at least implicitly assumed that T_2_ interference is mainly based on R_2_ activation in time to influence T_1_, not directly differentiating between S_2_−R_1_ and R_2_−R_1_ compatibility effects. The present study aims at addressing this gap—and at investigating whether the lack of evidence for R_2_−R_1_ effects in the PP paradigm in the reversed condition is only due to the low priority of the second task (i.e., responding to the flankers). We used the same conditions as in Experiment 1, with the same S-R rules for center (T_1_) and flanker (T_2_) letters in the matched condition, with opposite S-R rules for the flankers in the reversed conditions, and with separate letter sets and S-R rules in the neutral condition.

### Method

#### Participants

A fresh sample of 60 University of Otago psychology students (42 women, 15 men, three unknown) took part in the exchange for course credit.^2^ Participants ranged in age from 18 to 26 (*M* = 19.7) and they were predominantly right-handed (*M* = 59.7), as indexed by the Edinburgh Handedness Inventory (Oldfield, 1971). Additional six participants were also tested but excluded due to low accuracy (i.e., below 80%).

#### Apparatus, stimuli, procedure, and design

The apparatus, stimuli, procedure, and instructions were the same as in Experiment 1 except for the following changes. As mentioned above, the dual-task paradigm in which the flanker task was embedded was a PRP paradigm. Consequently, participants were asked to always first respond to the center letter and then subsequently respond to the outside letters. We omitted no-go stimuli from the experiment, as the PRP paradigm does not necessitate no-go-stimuli for either task, leaving four different letters in the matched and reversed conditions, and eight different letters in the neutral condition. Thus, participants had to give two responses on every trial. Omitting the no-go stimuli lead to fewer letters used for each participant. That is, in the matched and reversed conditions, four letters were used (i.e., both appearing as center and as flanker letters) for each participant; in the neutral condition, eight different letters (i.e., four as the center letters, four as the flanker letters) were used for each participant. Consequently, the number of trial types was also reduced to 12. In order to keep the experiment length similar to Experiment 1, each block had 64 trials. Each R_2_−R_1_ compatible trial type was presented eight times (as in Experiment 1, we excluded trials with the same stimulus for the center and the flankers) whereas each R_2_−R_1_ incompatible trial type was presented four times–resulting in 50% compatible and 50% incompatible trials.

### Results

Exclusion criteria for the analyses were the same as in Experiment 1. That is, practice blocks and the first experimental block were excluded from any further analysis as training. Trials in which any error was made were removed from the RT analyses (10.28%). None and 140 trials were removed from the analyses as RT outliers based on lower and upper RT cutoffs of 200 ms and 2 s, respectively.

#### Task 1: RT_1_ and PE_1_

##### Matched vs. reversed conditions

**Fig. 4:**
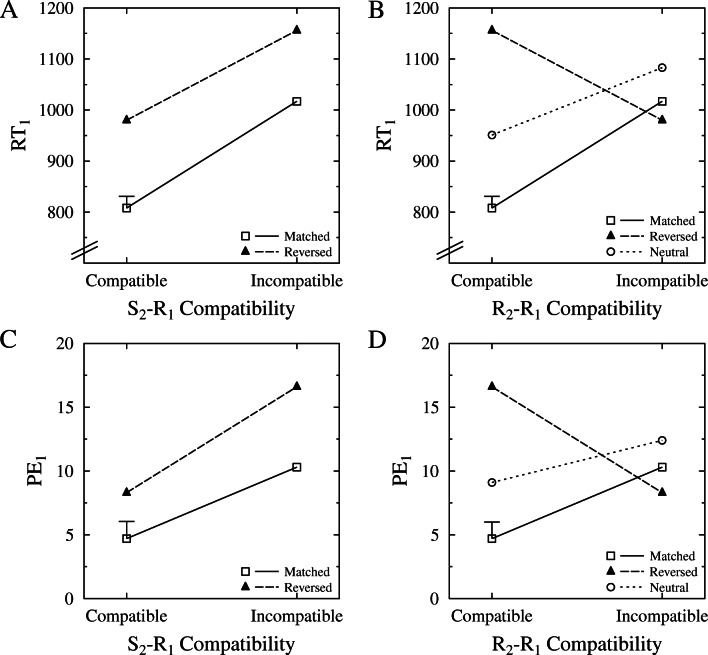
Experiment 2 results for the first task as a function of both stimulus (i.e., S_2_−R_1_) compatibility (A, C, for RTs and percent errors) and response (i.e., R_2_−R_1_) compatibility (B, D, for RTs and percent errors), separately for each condition. Please note that the figures on the right side (B,D) replot the data from the figures on the left (A,C) referring to a different type of compatibility. Error bar represents the pooled standard error. RT: reaction time, PE: percent errors, S: stimulus, R: response

Figure 4A shows the means of RT_1_ for the matched and reversed conditions as a function of S_2_−R_1_ compatibility. We ran an ANOVA with the between-subject factor condition (i.e., matched, reversed) and the within-subject factor of S_2_−R_1_ compatibility for RT_1_. The main effect of stimulus compatibility was again significant, *F*(1,38) = 189.29, *p* < .001, $\eta ^{2}_{p}=0.833$, with faster responses in stimulus compatible (894 ms) than in stimulus incompatible (1087 ms) trials. Moreover, there was a main effect of mapping condition, *F*(1,38) = 19.374, *p* < .001, $\eta ^{2}_{p}=0.338$, indicating faster responses in the matched condition (912 ms), than in the reversed condition (1068 ms). As in Experiment 1, the interaction of mapping condition and stimulus compatibility was not significant, *F*(1,38) = 1.412, *p* = .242, $\eta ^{2}_{p}=0.036$, indicating that the size of the stimulus compatibility effects was not modulated by condition (matched: 209 ms, reversed: 176 ms).

We again conducted parallel analyses on the percentage of erroneous responses for the first task (PE_1_). Figure 4C shows the means of PE_1_ for the matched and reversed conditions as a function of S_2_−R_1_ compatibility. This ANOVA revealed a significant main effect of stimulus compatibility, *F*(1,38) = 52.851, *p* < .001, $\eta ^{2}_{p}=0.582$, indicating fewer erroneous responses in stimulus compatible (4.3%) than in stimulus incompatible (8.3%) trials. The main effect of condition was significant, *F*(1,38) = 12.888, *p* = .001, $\eta ^{2}_{p}=0.253$, indicating fewer erroneous responses in the matched (7.5%) than in the reversed (12.5%) condition. The interaction of stimulus compatibility and mapping condition was not significant, *F*(1,38) = 1.850, *p* = .182, $\eta ^{2}_{p}=0.046$, indicating no differences in the stimulus compatibility effects between the matched (5.6%) and reversed (8.3%) conditions.

##### Neutral condition

Figure 4B shows the means of RT_1_ for all conditions (i.e., matched, reversed, neutral) as a function of R_2_−R_1_ compatibility, thus also showing the means of the neutral condition. Contrasting the findings of Experiment 1, we found a significant R_2_−R_1_ compatibility effect in the neutral condition, *F*(1,19) = 23.206, *p* < .001, $\eta ^{2}_{p}=0.55$, with faster R_2_−R_1_ compatible (951 ms) than on R_2_−R_1_ incompatible (1083 ms) trials, resulting in a 132 ms R_2_−R_1_ compatibility effect. Comparing this compatibility effect to the one obtained in the matched condition, we found a larger effect in the matched (209 ms) than in the neutral (132 ms) condition, *F*(1,38) = 5.329, *p* = .027, $\eta ^{2}_{p}=0.123$. Moreover, the compatibility effect obtained in the neutral condition did not differ significantly from the effect obtained in the reversed condition, *F*(1,38) = 1.566, *p* = .218, $\eta ^{2}_{p}=0.04$.

We again ran parallel analyses on PE_1_, and Figure 4D shows the means of PE_1_ for all three conditions as a function of R_2_−R_1_ compatibility. Again, we found a significant effect of R_2_−R_1_ compatibility in the neutral condition, *F*(1,19) = 8.729, *p* = .008, $\eta ^{2}_{p}=0.315$, with fewer erroneous responses in compatible (6.9%) than in incompatible (11.5%) trials. This compatibility effect did not differ in size with the one obtained in the matched condition, *F*(1,38) = 1.285, *p* = .264, $\eta ^{2}_{p}=0.033$. Comparing this effect to the compatibility effect obtained in the reversed condition, there was a significant interaction, *F*(1,38) = 5.995, *p* = .019, $\eta ^{2}_{p}=0.136$, with a larger effect in the reversed (8.3%) than in the neutral (3.6%) condition.

#### Task 2: RT_2_ and PE_2_

In Experiment 2, we conducted similar analyses for T_2_ as for T_1_. That is, we ran an ANOVA with the between-subject factor condition (i.e., matched, reversed, neutral) and the within-subject factor forward compatibility (i.e., compatible, incompatible second task stimulus to the first task stimulus). We again refer to stimulus compatibility here. As mentioned above, there is no possibility of stimulus compatibility in the neutral condition, therefore we always refer to response compatibility in this condition, generally labeling compatibility regarding the second task *forward compatibility*.

The ANOVA revealed a significant effect of forward compatibility, *F*(1,57) = 171.06, *p* < .001, $\eta ^{2}_{p}=0.75$, indicating shorter RT_2_s in the forward compatible trials (968 ms) than in the forward incompatible (1167 ms) trials. Moreover, we found a significant effect of condition, *F*(2,57) = 6.943, *p* = .002, $\eta ^{2}_{p}=0.196$. That is, RT_2_ was significantly shorter in the matched (974 ms) than in the reversed (1145 ms) condition (*p*<.001), as well as than in the neutral condition (1084 ms, *p* =.019). The difference between the reversed and the neutral condition was not significant (*p*=.267). The mapping condition x forward compatibility interaction was significant, *F*(2,57) = 3.673, *p* = .032, $\eta ^{2}_{p}=0.114$.

We conducted separate ANOVAs to further locate this interaction, always excluding one mapping condition at a time. The ANOVA including the matched and neutral conditions yielded a significant interaction, *F*(1,38) = 7.035, *p* = .012, $\eta ^{2}_{p}=0.156$, indicating a significantly larger forward compatibility effect in the matched (249 ms) than in the neutral condition (148 ms). The interactions of condition and forward compatibility when excluding the matched or neutral conditions were not significant (*p* =.177 and *p*=.180, respectively).

We ran a parallel analyses for the percentage of erroneous responses to the second task, PE_2_. This ANOVA revealed a significant effect of forward compatibility, *F*(1,57) = 59.467, *p* < .001, $\eta ^{2}_{p}=0.511$, indicating lower PE_2_ in forward compatible (7.4%) than forward incompatible (13.2%) trials. Moreover, the main effect of condition was significant, *F*(2,57) = 6.686, *p* = .002, $\eta ^{2}_{p}=0.19$. Pairwise comparisons revealed significant differences between the matched (7.5%) and the reversed (12.5%) conditions (*p*<.001), as well as between the matched and the neutral (10.9%) conditions (*p* = .015). The difference between the reversed and neutral conditions was not significant (*p*=.298). The interaction of forward compatibility and mapping condition just failed to be significant, *F*(2,57) = 3.099, *p* = .053, $\eta ^{2}_{p}=0.098$.

### Discussion

The results of Experiment 2 largely mirrored the findings of Experiment 1. That is, we found no interaction between the matched and reversed conditions’ S_2_−R_1_ compatibility effects, and the compatibility effect in the reversed condition was in the direction of an S_2_−R_1_ compatibility effect, and not based on an R_2_−R_1_ compatibility effect. The main difference to the findings of Experiment 1 is that we found evidence for a R_2_−R_1_ compatibility effect in the neutral condition. Thus, one cannot entirely discount the possibility that response compatibility does play a role as a BCE source—though evidently a lesser one than stimulus compatibility. We will return to this idea in the General Discussion. Moreover, the overall BCEs were descriptively much larger in the PRP paradigm (173 ms over all three mapping conditions) than in the PP paradigm (31 ms over all three mapping conditions), and this finding aligns well with the findings of Miller and Durst (2015) who also found smaller BCEs in the PP paradigm than in the PRP paradigm. In addition, Janczyk, Renas, and Durst (2018) also found that a task produces smaller BCEs when it does not always require a response, aligning well with the between-paradigm differences obtained in the present experiments. Whether or not a second response always has to be executed therefore seems to be important to the size of response compatibility-based BCEs.

Even though the overall compatibility effects were descriptively much larger, we still did not find evidence for direct R_2_ activation in the reversed condition. As in Experiment 1, in the reversed condition, the BCE was in the direction of an S_2_−R_1_ compatibility effect and did not differ in size from the S_2_−R_1_ compatibility effect found in the matched condition. It is interesting to note that in the reversed condition, R_1_ in a left-left response sequence was slower than in a left-right response sequence, and this must have been based on the S_2_−R_1_ compatibility in these trials. Because response grouping can possibly affect effects observed in first task RTs, we checked quite thoroughly whether the result pattern changed when requiring different minimum inter-response-intervals (IRIs) and found that response grouping did not affect the general result pattern.^3^

## General discussion

The present study aimed at disentangling stimulus and response compatibility as different sources of BCEs in dual-tasking paradigms using the same response sets for both tasks. To this end, we embedded the classic flanker task within two dual-task paradigms (i.e., the PP and PRP paradigms), assigning an S-R rule to the flanker letters that was either matched with respect to the center letter’s S-R rule (i.e., same S-R rule for the flankers as for the center letters), reversed (i.e., opposite S-R assignment for the flankers), or neutral (i.e., separate stimulus sets for the two tasks). Participants were always asked to respond first to the center letter (T_1_) and subsequently to the flanker letters in some trials (Experiment 1, responding only when the center letter required no response) or in all trials (Experiment 2).

The main results were rather consistent. Specifically, in both experiments, we found an S_2_−R_1_ compatibility effect in both the matched and the reversed conditions, with this effect changing little across the two mapping conditions, which suggests that R_2_−R_1_ compatibility had little effect. Thus, these results indicate that the BCE in these conditions was mainly driven by stimulus- rather than response-based compatibility. In the neutral condition, the results differed between the experiments: there was no R_2_−R_1_ compatibility effect for RTs in Experiment 1, and a 132 ms R_2_−R_1_ compatibility effect in Experiment 2. Thus, even though it might be a lesser role than the one played by stimulus compatibility, we cannot fully discount a role for response compatibility as a BCE source (see also Miller, 2006; Miller & Alderton, 2006; Ruiz Fernández & Ulrich, 2010).

### Implications for response selection

Regarding response selection, the most straightforward interpretation of the results obtained in the matched and reversed conditions is that only one S-R rule produced activation at a time. Specifically, it seems likely that S_2_ directly activated the corresponding T_1_ response based on the T_1_ S-R rule and thus created facilitation or interference relative to that rule, without activating R_2_ based on the T_2_ S-R rule. For instance, consider Experiment 2, where two overt responses were necessary on every trial: Here, R_1_ in the reversed condition was slower in same-hand response sequences (e.g., left-left) than in opposite-hand response sequences (e.g., left-right). If S_2_ had directly activated the corresponding response based on the second-task S-R rule, one would expect faster R_1_’s with same-hand response sequences (i.e., R_2_−R_1_ compatible) than with opposite-hand response (i.e., R_2_−R_1_ incompatible) sequences, just as they were in the matched and neutral conditions. This was not the case, however, so these results suggest that the BCEs observable in RT_1_ were driven by stimulus compatibility (i.e., an association between S_2_ and R_1_) rather than by preliminary activation of R_2_ by S_2_.

The stimulus-based compatibility effects in the matched and reversed conditions appear to be compatible with RSB accounts of dual tasking, because it seems as if only one S-R rule was active at a time—therefore potentially allowing for sequential response selection stages in the two tasks. However, the compatibility effects in these conditions do not seem to be consistent with the automatic response activation account of BCEs (e.g., Hommel, 1998), which was an initial extension to the classic bottleneck account of dual-task performance (e.g., Pashler, 1994). Specifically, if S_2_ activated R_2_ in time to influence T_1_ performance in accordance with the automatic activation account, then we should have observed a significantly decreased S_2_−R_1_ compatibility effect in the reversed condition compared to the matched condition. In fact, if R_2_ activation by S_2_ were the main source of the BCE, then the S_2_−R_1_ compatibility effect could even have been flipped in the reversed condition, with the combination of incompatible S_2_ stimuli and compatible R_2_ responses producing faster T_1_ responses than the reverse combination. Obviously, this did not happen.

However, we also have to consider the results obtained in the neutral condition. Here, the results differed between the two experiments—that is, we only found evidence for an effect of R_2_−R_1_ compatibility in the dual-task setting of the latter experiment. Nevertheless, the R_2_−R_1_ compatibility effect in Experiment 2 clearly shows the possibility of T_2_ interference independent from S_2_−R_1_ compatibility, and this is generally in line with earlier findings demonstrating that T_1_-irrelevant characteristics of T_2_ can still influence T_1_ performance (e.g., Miller & Alderton, 2006; Ruiz Fernández & Ulrich, 2010). Like many earlier BCEs, the neutral-condition R_2_−R_1_ compatibility effect seems to contradict pure RSB accounts of dual-task performance and to require an extension involving something like automatic response activation (Hommel, 1998).

As was argued above, the combined findings of the matched and reversed conditions seem to be inconsistent with the automatic response activation account, however, because the BCEs in these conditions were not based on S_2_ activating R_2_, but on S_2_ activating the corresponding R_1_ based on the T_1_ S-R rule. Using an extended PRP approach with three tasks that were less related than the two tasks in our present research, Janczyk et al., (2018) also argued that BCEs are located at the central response selection stage of processing. This idea would be consistent with the present conclusions, because we also argue that the combined results of the matched and reversed conditions contradict the idea of a separate stage generating response activation associated with a later task, and the findings in the neutral condition of Experiment 2 seem to contradict pure RSB accounts with non-overlapping response selection stages of the two tasks.

How can we then reconcile the present findings in all three mapping conditions and both experiments? For one thing, it might be necessary for models that aim at explaining BCEs to distinguish between situations with the same or different stimuli for the two tasks. Specifically, in situations with the same stimuli for T_1_ and T_2_, we see two closely-related accounts for the apparent absence of R_2_-based activations in the reversed condition. One possibility is that just one of the conflicting S-R rules can be active in working memory at a time. For example, while the first task is being performed, the second-task S-R rule might be fully inhibited in order to suppress any possible conflict. Such inhibition would not be needed when the two tasks used different stimuli, because there is no direct conflict in that case. Clearly, with only one active rule, S_2_ would only produce activation according to that rule, so S_2_ would not activate R_2_ and R_2_−R_1_ compatibility would have no effect. The other, closely related, possibility is that both S-R rules are active but that any given stimulus can only activate a single response, and the response that it activates is the one prescribed by the single most active S-R rule which is applicable to that stimulus. Thus, while working on T_1_ in the reversed condition, S_2_ would only produce activation according to the T_1_ S-R rule because this rule would be most active. Again, there would be no S_2_−R_2_ activation and hence no R_2_−R_1_ compatibility effect.

It is interesting that there was a between-experiment discrepancy with respect to the existence of a R_2_−R_1_ BCE in the neutral condition. Specifically, we found no R_2_−R_1_ compatibility effect in the PP paradigm where participants only had to respond overtly to T_2_ in one third of all trials (Experiment 1), but we did find an R_2_-R_1_ compatibility effect in the PRP paradigm where participants had to produce an R_2_ on every trial (Experiment 2). This discrepancy is probably explained by the greater relative T_2_ importance in the PRP paradigm, where it more often requires a response and would therefore have a higher priority (Miller and Durst, 2015). Furthermore, regarding other between-paradigm differences, Rieger et al., (2019) suggested that the coordination of two motor responses plays a crucial role in prioritizing tasks, which was only necessary in the PRP but not in the PP paradigm. Moreover, the extent to which T_2_ is processed online is different between the two paradigms (Mittelstädt and Miller, 2017), with stronger interference from T_2_ to T_1_ in the PRP paradigm than in the PP paradigm. Summarizing, differences in the characteristics of the two paradigms seem to be responsible for the existence of the BCE in the neutral condition in the PRP paradigm and its absence in the PP paradigm.

### Links to evidence from lateralized readiness potentials

Another way to study response activation in BCEs is to use lateralized readiness potentials (LRPs). Voluntary hand movements are preceded by an LRP (Deecke, Grözinger, & Kornhuber, 1976) which can be observed in electroencephalographic activity. This LRP is a reliable measure of hand-specific motor preparation (e.g., Osman, Moore, & Ulrich, 1995; Smulders & Miller, 2012). The onset of the LRP therefore provides a specific marker in time for the onset of hand-specific motor activation, and this marker can be used to subdivide the RT interval into subintervals before and after the onset of this motor activity (e.g., Hackley & Valle-Inclan, 1998; Miller & Ulrich, 1998). Experimental effects can thus be located to time intervals either before motor activation begins (i.e., stimulus-locked LRP effects), or after that point (i.e., response-locked LRP effects).

In LRP studies using the PRP paradigm (Ko & Miller, 2014) and the PP paradigm (Miller, 2017), both choice-related and no-go-related forms of the BCE seemed to be located prior to the onset of response activation (i.e., the BCE produces stimulus-locked LRP effects). Specifically, Ko and Miller (2014) addressed the question of whether the go/no-go selection-related processes of T_2_ influence RT_1_ before and/or after the onset of the LRP associated with R_1_. To this end, they used a letter classification task with manual response (i.e., left/right keypress) as T_1_ and a go/no-go foot press auditory task as T_2_. They found a no-go BCE which was located prior to T_1_ response initiation (i.e., stimulus-locked LRP effect). Similarly, Miller (2017) investigated the locus of the choice-related BCE in the PP paradigm. In this study, T_1_ was a letter classification task, T_2_ was a two-choice color classification task, and again the key finding was that the BCE affected the stimulus-locked LRP of T_1_. The LRP results of both studies thus suggest that BCEs arise because S_2_ influences T_1_ response selection rather than because it activates the corresponding R_2_ with which it is associated, much in line with our present findings.

### Links to action effect based compatibility effects

The present evidence that stimulus compatibility is more critical to the BCE than response compatibility also fits well with research on action effect compatibility (e.g., Janczyk et al., 2014). According to ideomotor theory (see for example, Hommel, Müsseler, Aschersleben, & Prinz, 2001; Pfister & Janczyk, 2012; Stock & Stock, 2004), responses and other actions are cognitively coded in terms of their perceived effects, so they are faster when the action effects are compatible with the actions themselves (i.e., action-effect compatibility effects; e.g., Kunde, 2001 Kunde, Hoffmann, & Zellmann, 2002; Pfister, Kiesel, & Melcher, 2010). For example, left- and right-hand responses are faster when they produce lights appearing on the same side (i.e., left vs. right) than when they produce lights that appear on the opposite side.

Action-effect compatibility effects have also been observed in dual-task paradigms similar to those used in the present studies. For example, Janczyk et al., (2014) used a dual-task paradigm resembling a PRP task, and in different conditions R_2_ could lead to either a compatible (e.g., light appearing on the same side as the response) or incompatible (e.g., light appearing on the opposite side to the response) action effect. In blocks with incompatible action effects, they found that the standard R_2_−R_1_ BCE disappeared—and descriptively even reversed—suggesting that response compatibility was less important than action effect compatibility. As argued above, our findings indicate that response compatibility is also less important than stimulus compatibility. Together, these results raise the question of whether response compatibility is rather low in a hierarchy of potential BCE sources. Taking the action effect account to an extreme, in fact, one could even argue that any case of response compatibility is really only based on action effect compatibility, because any response in a typical multitasking paradigm also has at least the effect of generating small proprioceptive feedback from emitting that response on the keyboard. These kinds of effects obviously cannot be completely disentangled from making the actual response itself, making it difficult to be sure whether the BCE is driven by the activation of the response or the expected action effect.

### Conclusion

We embedded the classic flanker task in two different dual-task paradigms to separate the contributions of second-task stimulus (S_2_−R_1_) and response (R_2_−R_1_) compatibility to BCEs. To this end, we varied the S-R rule between-subjects to be either matched, reversed, or neutral. The results across both experiments suggest that the BCE is mainly driven by stimulus compatibility (i.e., S_2_−R_1_) rather than by response compatibility (i.e., R_2_−R_1_), because the BCEs in both the matched and reversed conditions were in the direction of S_2_−R_1_ compatibility effects—with little change in this effect across mapping conditions. It thus seems that when the T_1_ S-R rule is applicable to S_2_, this rule (and consequently, the resulting stimulus compatibility) is what drives the BCE, rather than R_2_ activation generated by applying the T_2_ S-R rule to S_2_, contrary to some previous theoretical accounts of the BCE. This stimulus compatibility-based BCE seems generally consistent with RSB models of dual tasking, and seems generally incompatible with the extension of automatic response activation. However, the results obtained in the second experiment’s neutral condition (i.e., evidence for R_2_−R_1_ compatibility) would need such an extension, and it might be therefore necessary to distinguish situations with the same or different stimuli for the two tasks.

## Footnotes

1 In order to check whether the null-effect was based on individual differences in compatibility effects (i.e., participants with opposing compatibility effects negating an overall significant effect of R_2_−R_1_ compatibility in the neutral condition), we used the methods described in Miller and Schwarz (2018). Specifically, we analyzed whether the individual variation was significantly larger than one would expect from the trial-to-trial variation. This was not the case, *F*(1,19) = 1.183, *p*=.262. Thus, it seems fair to conclude that there was little or no R_2_−R_1_ compatibility effect on RT in the neutral condition.

2 Note that due to technical issues, demographic data from three participants are missing. The data reported here refer to the participants where the information is available.

3 Specifically, we ran parallel analyses excluding any responses where the IRI was smaller than 100, 150, or 200 ms and checked whether the result pattern changed. This was generally not the case. As an additional check, we also conducted a median-split within the IRIs of each participant, and added the factor short vs. long IRIs into the ANOVA conducted on the RTs, to check whether any effect is modulated by response grouping. Again, we did not find any evidence that response grouping was responsible for the reported results.
